# The efficacy and safety of targeted therapy plus fulvestrant in postmenopausal women with hormone-receptor positive advanced breast cancer: A meta-analysis of randomized-control trials

**DOI:** 10.1371/journal.pone.0204202

**Published:** 2018-09-20

**Authors:** Gao Chanchan, Su Xiangyu, Shi Fangfang, Chen Yan, Gu Xiaoyi

**Affiliations:** Department of Oncology, Zhongda Hospital of Southeast University, Nanjing, China; Universita degli Studi di Verona, ITALY

## Abstract

**Objective:**

To evaluate the efficacy and safety of targeted therapy plus fulvestrant for postmenopausal patients with hormone receptor-positive advanced breast cancer.

**Methods:**

Pubmed, Embase and Web of Science databases were systematically searched on February 26, 2018. Eligible studies were screened according to selection criteria, and two reviewers independently extracted outcome data which included progression-free survival, overall survival, objective response rate, clinical benefit rate and toxicities. RevMan 5.3 and STATA 11.0 software were used to conduct meta-analysis.

**Results:**

Thirteen articles including twelve randomized-control trials fulfilled selection criteria. There was no evidence regarding the existence of publication bias and high-risk bias of quality in the selected studies. In previously endocrine therapy-treated postmenopausal patients with hormone-receptor positive advanced breast cancer, the PFS (HR = 0.77, 95%CI: 0.66–0.91) and ORR (RR = 1.78, 95%CI: 1.35–2.34) of combination therapy group were significantly higher than that from fulvestrant monotherapy group. Besides, a statistically significant difference in PFS was found across the two arms in postmenopausal women with PIK3CA-mutant ctDNA tumor (HR = 0.52, 95% CI: 0.39–0.69). Moreover, the risk of adverse events (RR = 1.09, 95%CI: 1.05–1.13), CTCAE≥3 (RR = 1.97, 95%CI: 1.49–2.60) and discontinuation due to adverse events (RR = 4.91, 95%CI: 3.37–7.15) were also significantly different between two treatment groups. Sensitivity analysis showed PLOMA-3 trial was an important factor of heterogeneity.

**Discussion:**

Even though the combination of targeted therapy plus fulvestrant improved PFS and increased ORR in advanced breast cancer patients, the toxicities of combination therapy were also higher than fulvestrant monotherapy. Further studies related to inhibitors targeting the specific signaling pathway or receptors are urgently needed, and more efforts concerning precision medicine of targeted therapy plus endocrine therapy should be taken to improve the clinical benefits.

## Introduction

Breast cancer is the most common cancer in women worldwide[1], it estimates that one in eight to ten women might suffer from this malignancy during her lifetime[2]. Early breast cancer is believed to a potential curable disease, and the appropriate treatments include breast-conserving surgery, radiotherapy and neoadjuvant endocrine/chemotherapy therapy. A meta-analysis conducted by Early Breast Cancer Trialists’ Collaborative Group suggests that after breast conservation, radiotherapy could effectively reduce the 10-year risk of recurrence (RR = 0.52, 95% CI: 0.48–0.56) and the 15-year risk of death (RR = 0.82, 95% CI: 0.75–0.90)[3]. However, advanced breast cancer (ABC, locally advanced or metastatic breast cancer) are incurable where the goals of treatments are prolongation of survival and maintaining the quality of life. It has been documented that, postmenopausal women with hormone-receptor positive (HR+), human epidermal growth factor receptor type2-negative (HER2-) tumors represent the majority of advanced breast cancer patients[4, 5]. International guidelines recommend endocrine therapy (tamoxife, anastrozole, letrozole, exemestane and fulvestrant, etc) are the first-line treatment while these incurable patients don’t have immediately life-threatening disease[6, 7].

Fulvestrant, an analog of 17-beta estradiol, is the first-generation selective estrogen receptor downregulator (SERD), which is approved for the treatment of HR+ postmenopausal patients. Fulvestrant binds to the estrogen receptor and makes it more hydrophobic, resulting in its accelerated degradation[8]. For postmenopausal ABC patients, several studies indicates that fulvestrant is at least as effective as other endocrine therapies[9, 10], and adverse events of patients treated with fulvestrant is usually mild or moderate, including nausea, injection site reactions, weakness, and elevated transaminases, etc[11, 12].

However, for treatment of advanced breast cancer, intrinsic or acquired endocrine resistance are major obstacle in achieving better clinical outcomes[13]. And the possible mechanisms of endocrine resistance involves alterations to the ER and its co-regulators, key cell cycle checkpoints, cell survival pathway and apoptosis, overexpression and/or amplification of growth factor, etc[14, 15]. The intensive efforts to overcome this resistance led to the development of combination therapies which also include targeted agents plus endocrine therapy, such as everolimus plus exemestane [16] and palbociclib plus fulvestrant[17]. Herein, we conduct a meta-analysis of randomized-controlled trials (RCTs) to quantitatively assess the efficacy and toxicities of targeted therapy plus fulvestrant in postmenopausal women with hormone-receptor positive advanced breast cancer.

## Materials and methods

### Search strategy

Electronic databases including Embase, Pubmed and Web of Science were systematically searched on February 26, 2018. The key search terms were selective estrogen receptor downregulator OR fulvestrant OR faslodex, breast cancer OR breast neoplasm OR breast carcinoma OR breast malignancy. No language restriction was used during the literature search. The bibliography of relevant studies, reviews, and conferences were manually searched.

### Selection criteria

The following inclusion criteria were applied for subsequent analysis: (1) randomized-controlled trial; (2) postmenopausal women with hormone receptor-positive (estrogen-receptor positive and/or progesterone-receptor positive) advanced breast cancer; (3) studies about targeted therapy plus fulvestrant (the intervention group) and fulvestrant alone(the comparator); (4) at least one of efficacy or tolerability index was sufficiently reported. Efficacy was chosen as the primary outcome, including progression-free survival (PFS), overall survival (OS), overall response rate (ORR) and clinical benefit rate (CBR, best overall response of complete response, partial response, or stable disease ≥24 weeks). Toxicity was chosen as the secondary outcome, which contained adverse events, sever adverse events, discontinue and National Cancer Institute Common Terminology Criteria for Adverse Events (CTCAE) ≥3. Moreover, only the most recent or detailed study was selected for duplicate publication.

### Data extraction and quality assessment

Two investigators (Gao CC and Su XY) independently reviewed articles and extracted data, the following information were acquired from each eligible study: first author, publication time, study design, setting, follow-up, characters of participants, interventions, efficacy and toxicity. For time-to-event outcomes (PFS and OS), we extracted hazard ratio (HR) and 95% confidence interval (CI) as treatment effect. For HR and 95%CI data that could not be directly extracted from the main text and supplementary materials, they were obtained indirectly from published Kaplan-Meier curves using the Tierney’s method[18]. For dichotomous outcomes (ORR, CBR and toxicity), we extracted the number of patients who had relevant events and total number of patients, the risk ratio (RR) with 95%CI was expressed as treatment effect.

To measure the bias risk, two reviewers (Gao CC and Su XY) independently assessed the quality of each study according to the Cochrane Collaboration criteria, which includes the following seven points: random sequence generation, allocation concealment, blinding of participants and personnel, blinding of outcome assessment, incomplete outcome data, selective reporting and other bias. Any disagreements during extraction and quality assessment were resolved by consensus.

### Statistical analysis

We estimated heterogeneity using the Cochran’s Q and the I^2^ statistic. When *P*<0.10 or I^2^>50%, a random-effect model was selected to pool effect size, otherwise, a fixed-effect model was used. Egger’s test and Begg’s test were used to assess the potential publication bias, and *P*<0.10 indicated statistical significance. Sensitivity analysis was performed by step-wise removal of single study. STATA software 11.0 and RevMan 5.3 software were used to perform statistical analyses.

## Results

### Characteristics of included studies

**Fig 1:**
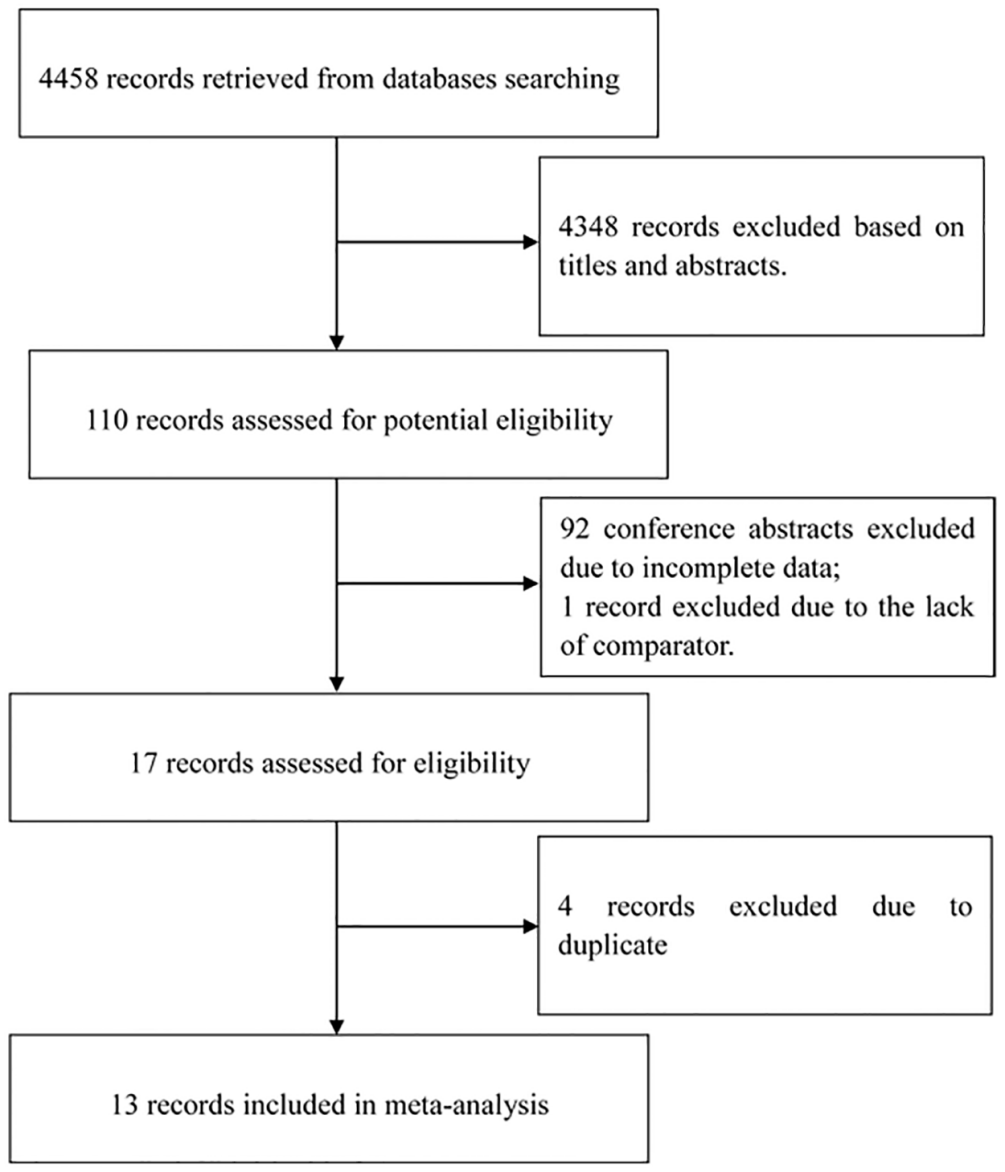
Flow chart of the included study.

The primary databases search yielded 4458 relevant studies. After screening titles, abstracts and full texts, 4445 records were excluded, and 13 studies that contained 12 RCTs met our inclusion criteria (Fig 1)[19–31]. Two articles reporting PLOMA-3 trial[25, 28] were selected to calculate relevant efficacy and safety endpoints in postmenopausal patients. Each single article evaluated proteasome inhibitor in combination with fulvestrant[24], IGF inhibitor in combination with fulvestrant[20], EGFR inhibitor in combination with fulvestrant[21], MAPK inhibitor in combination with fulvestrant[23] and FGFR inhibitor in combination with fulvestrant[29]. Two articles assessed VEGF inhibitors in combination with fulvestrant[19, 22], three articles reported CDK4/6 inhibitors in combination with fulvestrant[25, 28, 30], and three articles assessed PI3K inhibitors in combination with fulvestrant[26, 27, 31]. Subjects of eleven RCTs with twelve articles were confirmed to ET-resistant. More details concerning characteristics of included studies were shown in Table 1.

**Table 1:** Characteristics of included studies.

Study	Design	Setting	Schedule	Follow–up	Patients
Hyams[19]	Phase II, 1:1 RCT	19 centres in Australia, Brazil and the USA	Cediranib [oral, 45 mg/day]+FUL [LD](n = 31) VS Placebo+FUL(n = 31)	Recrument: March 2007-April 2008; cut-off: 12 December 2008.	HR+/HER unknown postmenopausal patients with evaluable disease whose disease had progressed on prior hormonal therapy. Age: mostly 18–65 yr. combined therapy VS comparator: Prior ET Tamoxifen 24/31 VS 24 /31; Letrozole 11/31 VS 15 /31;; Anastrozole 12 /31 VS 10 /31 Exemestane 7/31 VS 6/31; Measurable disease: 18/31 VS 12/31.
Robertson[20]	Double-blind, phase II, 2:1 RCT	58 centres in the USA, Europe, Canada, and Australia.	Ganitumab [IM, 12mg/kg day 1, 15/ 28 days] +FUL [LD]/ exemestane[25mg/day] (n = 106) VS FUL/exemestane (n = 50)	Recrument: March 2008 -July 2009; cut-off: September 2011.	HR+ postmenopausal patients with endocrine-resistant or recurrent breast cancer. combined therapy VS comparator: Median age: 61yr VS 62yr. HER+: 7/106 VS 1/50. FUL treatment during study: 72/106 VS 34/50.
Clemons[22]	Double-blind, multicentre, phase II, 1:1 RCT	13 Canadian cancer centres	Vandetanib[100 mg/day]+ FUL [HD](n = 61) VS Placebo +FUL(n = 68)	Recrument: October 2009- October 2011; cut-off: July 2013.	HR+, endocrine-resistant postmenopausal patients with bone metastases. combined therapy VS comparator: Mean age: 61.6yr VS 57.7yr. HER+: 3/61 VS 1/68. Prior ET: Tamoxifen/AI treatment 42 /61 VS 53/68; Tamoxifen/AI adjuvant treatment 9/61 VS 10 /68. Measurable disease: 21/61 VS 40/68. Metastatic disease: Liver 14/61 VS 23 /68; Lung 12/61VS22/68; Lymph node 14/61 VS 16/68; Skin 3 /61 VS 0 /68.
Burstein[21]	Double-Blind, Phase III, 1:1 RCT	NA	Lapatinib[oral, 1500mg/day]+ FUL[LD] (n = 146) VS Placebo+ FUL (n = 145)	Recrument: September 2006; cut-off: June 2010.	HR+ postmenopausal patients. combined therapy VS comparator: Age: mostly 40-69yr. HER+: 24/146 VS 30/145. Prior ET treatment: tamoxifen 83/146 VS 82/145; AI 141/146 VS 140/145.
Zaman[23]	Double-blind, multicentre, phase II, 46:43 RCT	20 centres in Switzerland and Belgium	Selumetinib[oral, 75mg×2/day] + FUL[HD] (n = 23) VS Placebo+ FUL (n = 22)	Recrument: November 2010-March 2012; Median follow-up: 22 months.	HR+/HER2-, postmenopausal patients whose disease had progressed after AIs-treatment. combined therapy VS comparator: median age: 66yr VS 69yr. Prior TAM treatment: 14/22 VS 11/20. Visceral metastases: 13/22 VS 11/20. Measurable disease: 15/22 VS 15/20.
Cristofanilli[25]and Loibl[28]	Double-blind, multicentre, phase III, 2:1 RCT	144 centres in 17 countries	Palbociclib [oral, 125 mg/day for 3 weeks, followed by a week off in a 28-day cycle] + FUL[HD] (n = 345) VS Placebo+ FUL (n = 172)	Recrument: October 2013-August 2014; Cut-off: March 2015.	HR+/HER2- female patients whose disease had progressed after previous endocrine therapy. combined therapy VS comparator: median age: 57yr VS 56yr. postmenopausal: 275/345 VS 138/172. measurable disease: 268/345 VS 138/172. Prior ET: first-line 160/345 VS 91/172; second-line 140/345 VS 61/172.
Krop[26]	double-blind, phase II, 1:1/1:2 RCT	123 medical centres in 21 countries	Part1: Pictilisib[oral, 340mg/day]+ FUL[HD](n = 89) VS Placebo+FUL (n = 79) Part 2: Pictilisib [oral, 260mg mg/day] +FUL[HD] (n = 41) VS Placebo+FUL (n = 20)	Part 1: Recrument: September 2011-January 2013; Median follow-up: 17.5 months. Part 2: Recrument: March 2013-January 2014; Median follow-up: 12.9 months.	Part 1: HR+/HER2- postmenopausal patients with AIs resistance. combined therapy VS comparator: median age: 60yr VS 63yr. measurable disease: 51/89 VS 43/79. visceral metastases: 51/89 VS 42/79. PIK3CA mutation positive: 38/89 VS 32/79. Part 2: HR+ /HER2-, AIs-resistant postmenopausal patients with PIK3CA mutation. combined therapy VS comparator: median age: 58yr VS 63yr. measurable disease: 29/41 VS 13/20. visceral metastases: 21/41VS 10/20.
Adelson[24]	Open-label, multicenter, phase II, randomized trial	NA	Bortezomib[intravenous infusion, 1.6 mg/m^2^ at day 1, 8, 15/28 days]+ FUL[500mg IM days 14, 1, 15 and then day 1/each 28 days] (n = 57) VS FUL (n = 59)	Recrument: June 2010-October 2013; Cut-off: NA.	ER+/HER2-, AIs-resistant postmenopausal patients. combined therapy VS comparator: Age: 57yr VS 59yr. Metastatic sites: Bone 46/57 VS 45 /59; Lung 9/57 VS 23/59; Liver 22/57 VS 21/59.
Baselga[27]	Double-blind, multicenter, phase III, 1:1 RCT	267 centres in 29 countries	Buparlisib [oral, 100 mg/day]+FUL[HD](n = 576) VS Placebo + FUL (n = 571)	Recrument: September 2012-September 2014; cut-off: April 2015.	HR+/HER2-, AIs-resistant postmenopausal patients. combined therapy VS comparator: Age: 62yr VS 61yr. PI3K pathway status in tumor tissue: Activated 188/576 VS 184/571; Non-activated 239/576 VS 240/571; Unknown or missing 149/576 VS 147/571.
Musolino[29]	Double-blind, multicenter, phase II, 1:1 RCT	36 centers in 12 contries	Dovitinib [oral, 500 mg/5 day one week]+FUL[IM, 500mg/each 2 weeks](n = 47) VS Placebo+FUL (n = 50)	Recrument: May 2012 -November 2014; cut-off: April 2015.	HR+/HER2-, ET-resistant postmenopausal patients. combined therapy VS comparator: Age: 62yr VS 61yr. FGF pathway amplified: 15/47 VS 17/50. Metastatic site: Bone 39/47 VS 36 /50; Lymph nodes 21/47 VS 26/50; Liver 22/47 VS 16/50. Prior ET: Tamoxifen 27 /47 VS 21 /50; Letrozole 18 /47 VS 23/50; Anastrozole 16/47VS 18/50; Exemestane 8/47 VS 9/50.
Sledge[30]	Double-blind, phase III, 2:1 RCT	142 centers in 19 countries.	Abemaciclib[oral, 150 mg×2/day]+FUL[HD](n = 446) VS Placebo+FUL(n = 223)	Recrument: August 2014-December 2015 cut-off: February 2017.	HR+/HER2-, ET-resistant patients. combined therapy VS comparator: Age: 59yr VS 62yr. Postmenopausal patients: 371/446 VS 180/223. Measurable disease: 318/446 VS 164/223. Prior AI: 316/446 VS 149/223. Metastatic site: Visceral 245/446 VS 128 /223; Bone only 123 /446 VS 57 /223.
Leo[31]	Double-blind, multicentre, phase III, 2:1 RCT	200 centres in 22 countries	Buparlisib[oral, 100 mg/day]+FUL[HD](n = 289) VS Placebo+FUL(n = 143)	Recrument: January 2013- March 2016; cut-off: May 2016.	HR+/HER2-, postmenopausal patients who had relapsed on or after endocrine therapy and mTOR inhibitors. combined therapy VS comparator: Age: 60yr VS 62yr. mTOR inhibitor: everolimus 286/289 VS 142 /143; ridaforolimus 3/ 289 VS 1/143. Metastatic sites: Bone219 /289 VS 111/143; Visceral 212 /289 VS 103/143; Liver 137 /289 VS 76/143.

LD: loading does, 500 mg IM on day 1, and 250 mg on days 15, 29 and every 28 days thereafter;

HD: high does, 500 mg IM days 1, 15, 29 and every 28 days afterwards; IM: intramuscular injection; FUL: fulvestrant.

### Publication bias and quality assessment

Egger’s test and Begg’s test were used for research endpoints with more than two articles. There was no obvious evidence of publication bias in the selected studies (*P*> 0.10).

**Fig 2:**
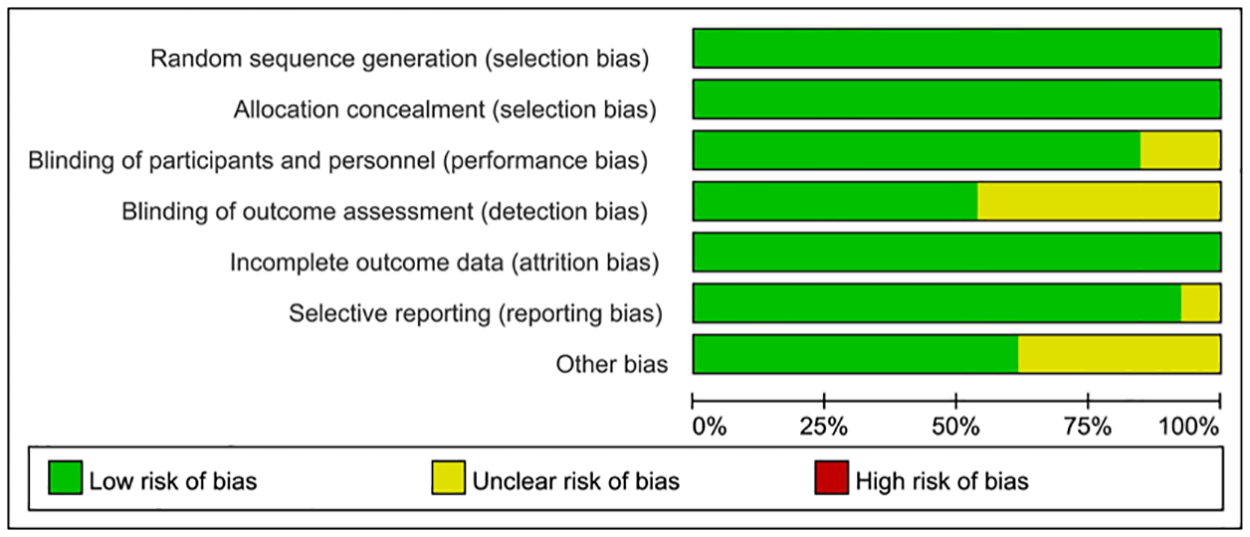
Risk of bias graph: Review authors’ judgements about each risk of bias item presented as percentages across all included studies.

**Fig 3:**
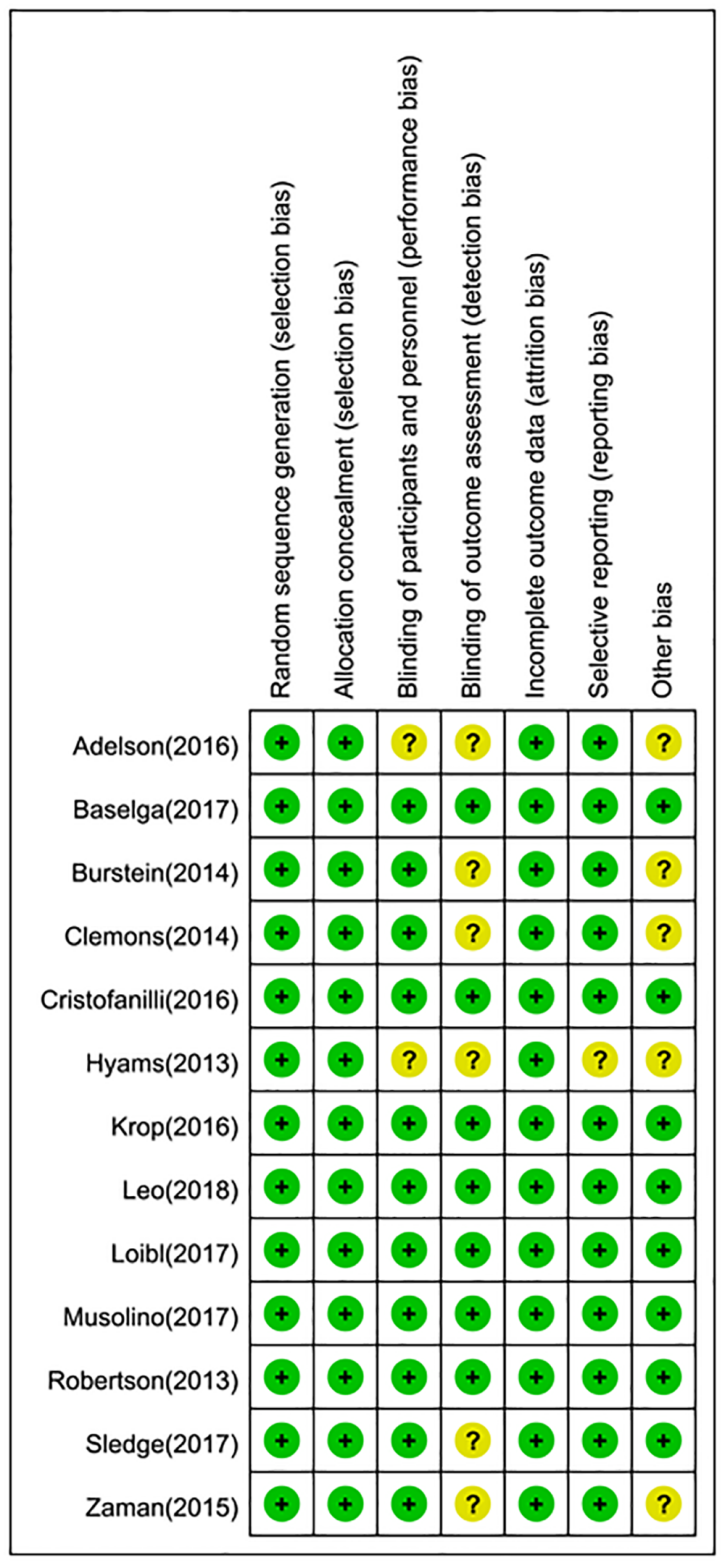
Risk of bias summary: Review authors’ judgements about each risk of bias item for each included study.

The quality assessments of included studies were summarized in Figs 2 and 3. The overall risk of bias was low, particularly in the selection bias and attribution bias. For blinding, one trial was an open-label[24], the other one[19] did not describe relevant information, these two articles were classified as unclear risk. Furthermore, most outcome assessments were blind and judged to be at low risk. For reporting bias, Hymas et al[19] only reported ORR of patients with measurable disease and did not report the ORR of total population, therefore, this trial was judged to be at unclear risk, other trials were judged at low risk.

### Efficacy

#### Progression free survival

**Fig 4:**
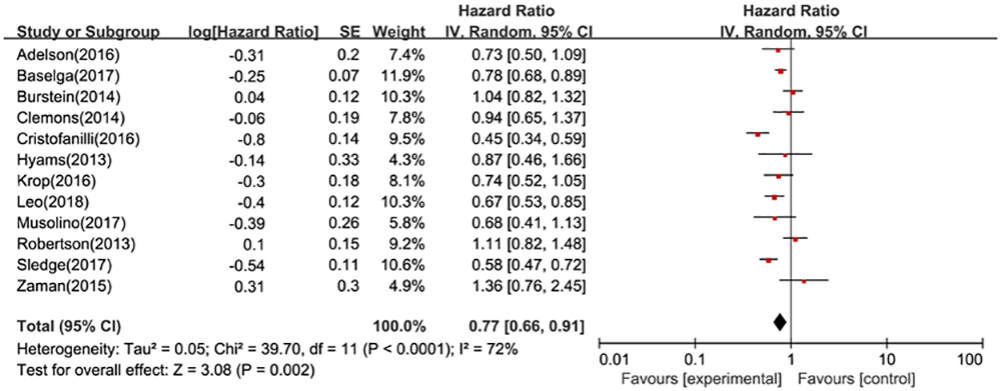
Forest plot for progression free survival in postmenopausal patients with HR+ advanced breast cancer.

Data on hazard ratios of PFS were available in all 12 RCTs. The random-effect model (*P*<0.0001, I^2^ = 72%) showed that pooled HR was 0.77(95%CI: 0.66–0.91) (Fig 4). Besides, there were nine trials provide PFS data for postmenopausal women with HR+, HER2- advanced breast cancer, the pooled HR of PFS determined by the random-effect model (*P* = 0.004, I^2^ = 72%) was 0.71(95%CI: 0.60–0.85). Moreover, nine articles clearly indicated the participants were endocrine-therapy resistant, the pooled effect size was 0.76 (95%CI: 0.63–0.92).

PFS data from patients with measurable disease at baseline were obtained from 2 trials and did not show a significant difference between two treatment arms (HR = 0.88, 95%CI: 0.55–1.40).

The relationship between PIK3CA mutation and clinical benefit of combination regimen remained unclear. There were three studies reported PFS concerning PIK3CA mutant status in archival or newly collected tumor issue, random-effect model (*P* = 0.07, I^2^ = 57%) showed that there was not statistically significant difference in PFS between combination therapy and the comparator (HR = 0.70, 95%CI: 0.48–1.02). Besides, two trials of above three studies also reported data about PIK3CA mutation detected in circulating tumor DNA(ctDNA), and fixed effect model (*P* = 0.50, I^2^ = 0%) indicated that the combination therapy had longer PFS than fulvestrant monotherapy among patients with PIK3CA-mutated ctDNA (HR = 0.52, 95%CI: 0.39–0.69).

#### Overall survival

Most included studies did not have mature overall survival data at the cut-off date, the HR was only found in two trials, and no statistically significant difference was observed between treatment agents(HR = 0.88, 95%CI:0.67–1.17).

#### Overall response rate

**Fig 5:**
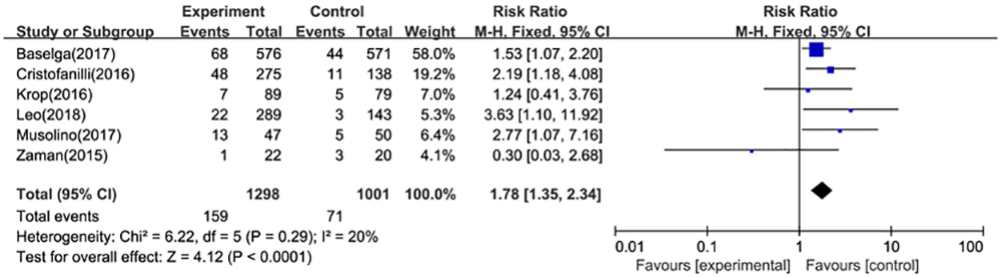
Forest plot for overall response rate in postmenopausal patients with HR+, HER2- advanced breast cancer.

Six articles including 2299 participants with HR+/HER2- tumor reported ORR data, the pooled RR was 1.78(95%CI:1.35–2.34) by using the fixed effect model (*P* = 0.29, I^2^ = 20%) (Fig 5). Besides, there were also three studies represented data related to patients with measurable disease. The fixed effect model (*P* = 0.98, I^2^ = 0%) indicated the combination therapy significantly improve overall response rate (RR = 2.35, 95%CI: 1.35–4.11).

#### Clinical benefit rate

**Fig 6:**
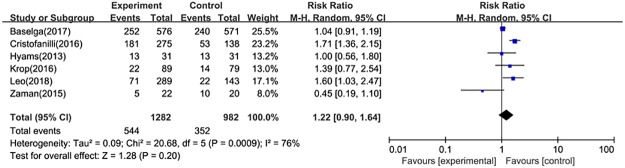
Forest plot for clinic benefit rate in postmenopausal patients with HR+, HER2- advanced breast cancer.

CBR data were extracted from six trials, which included 2264 subjects. We did not observe a significant difference between the intervention arm and the comparator (HR = 1.22, 95%CI: 0.90–1.64) (Fig 6). Two studies presented relevant information in patients with measurable disease, the pooled RR was 1.15(95%CI: 0.68–1.94).

### Tolerability

#### Adverse events

Data of adverse events were available for 4 RCTs. the results of fixed-effect model (*P* = 0.13, I^2^ = 46%) showed that the pooled RR was 1.09 (95%CI: 1.05–1.13).

#### Sever adverse events

**Fig 7:**
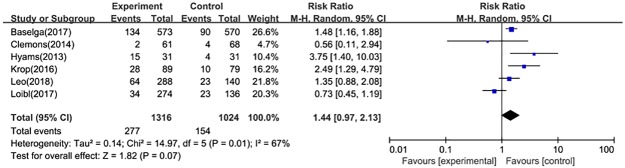
Forest plot for sever adverse events in postmenopausal patients with HR+ advanced breast cancer.

There was no significant difference in the total incidence of sever adverse events (SAEs) between two treatment groups (RR = 1.44, 95%CI: 0.97–2.13) (Fig 7). Additionally, three clinical trials reported SAEs related to treatment drugs, and the fixed-effect model (*P* = 0.16, I^2^ = 45%) showed that the pooled RR was 4.23(95%CI: 1.62–11.03).

#### CTCAE≥3

**Fig 8:**
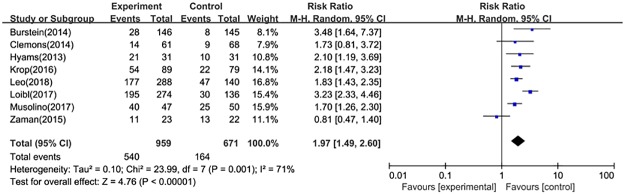
Forest plot for CTCAE≥3 in postmenopausal patients with HR+ advanced breast cancer.

Data concerning CTCAE≥3 was reported in eight studies. The results of random-effect model (*P* = 0.001, I^2^ = 71%) indicated that the combination therapy was associated with significantly greater risk of CTCAE≥3 (RR = 1.97, 95%CI: 1.49–2.60) (Fig 8).

#### Discontinuation

**Fig 9:**
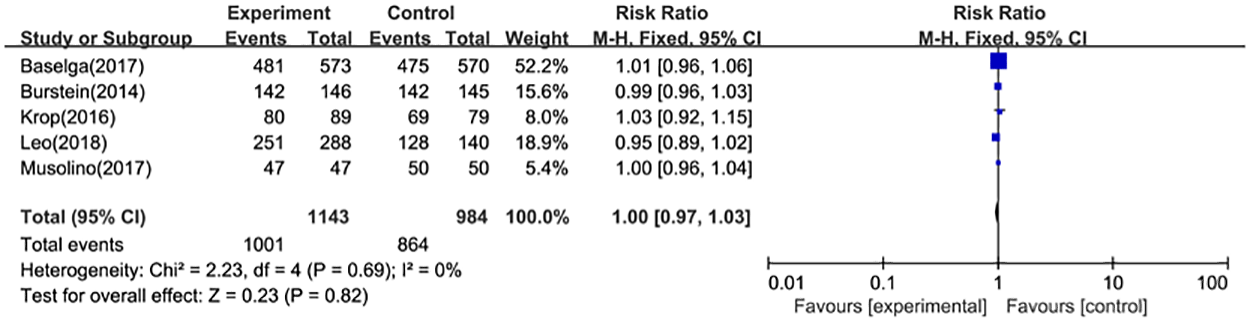
Forest plot for discontinuations in postmenopausal patients with HR+ advanced breast cancer.

**Fig 10:**
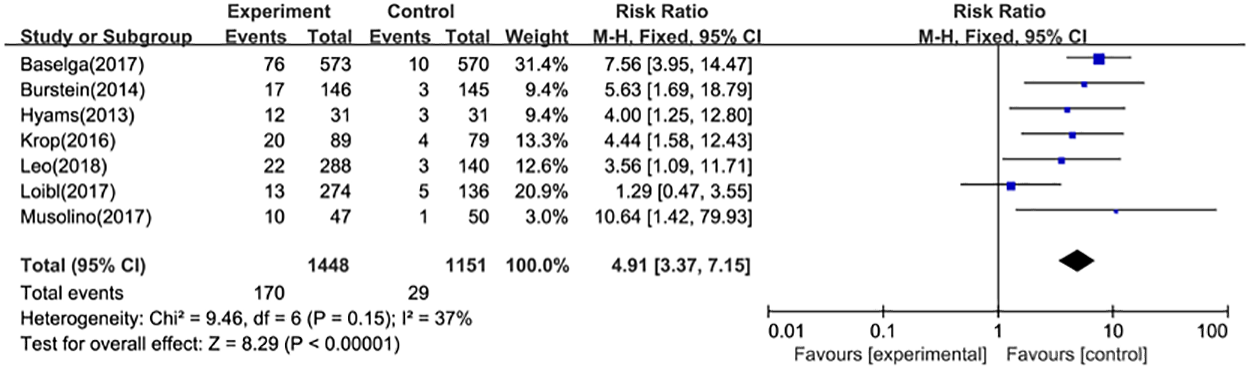
Forest plot for discontinuations due to adverse events in postmenopausal patients with HR+ advanced breast cancer.

The reasons for discontinuation include death, disease progression, adverse events, loss to follow-up, non-compliance to study treatment, physician decision, participant or guardian decision, protocol deviation, termination of the study by sponsor, technical problems. Data on discontinuation were available for five RCTs. The pooled RR was 1.00 (95%CI: 0.97–1.03) (Fig 9). Furthermore, seven studies reported data on treatment discontinuation due to AEs. The estimate was significantly different between two treatment arms (RR = 4.91, 95%CI: 3.37–7.15) (Fig 10).

### Sensitivity analysis

For efficiency index, removal of the second period trial of krop et al, combination therapy had significant benefit for PFS in patients with PIK3CA mutation in tumor tissue (HR = 0.64, 95%CI: 0.4–0.98). Other effect size of PFS had not been significantly altered by any included trials, while there was one trial (PLOMA-3) obviously contributing heterogeneity. Sensitivity analysis concerning ORR and CBR demonstrated that pooled RR were not significantly changed by excluding individual study stepwise, but the value of I^2^ for ORR decreased to 0% after removing the study conducted by Zaman et al.

For toxicity index, removal of Leo’s or Musolino’s study, the confidence interval of RR related to AEs would contain the number 1; in addition, removal of studies written by Clemons or Loibi, the pooled effect size demonstrated the incidence of SAEs was associated with targeted therapy plus fulvestrant (removal of Clemons’s study: pooled RR = 1.51, 95%CI: 1.01–2.26; removal of Loibi’s study: pooled RR = 1.57, 95%CI: 1.29–1.90). Moreover, PLOMA-3 was an important factor to heterogeneity of tolerability index, and Zaman et al’s article was also contributed to heterogeneity of CTCAE≥3.

## Discussion

Endocrine therapy is a preferred approach for patients with HR-positive advanced breast cancer[32]. Some clinical studies have shown that estrogen antagonist and aromatase inhibitor can improve survival time and decrease mortality rate of HR-positive advanced breast cancer patients. However, side-effect and drug resistance hampered the long-term use of above two kinds of treatments[33–36]. Fulvestrant has become a new choice for advanced breast cancer with its unique mechanism of action, and it has been recommended as first-line treatment of postmenopausal women with advanced breast cancer[7]. Initially, patients should receive once-monthly intramuscular injections of fulvestrant 250 mg. CONFIRM trial demonstrated that, fulvestrant 500mg provided a significant improvement in both PFS and OS without an increasing in the toxicity compared with fulvestrant 250mg[12, 37]. Therefore, fulvestrant 500mg was adopted as the preferable dose.

To improve the therapeutic efficacy and overcome resistance, fulvestrant has been evaluated in combination with other endocrine agents or novel targeted drugs[8]. Regarding fulvestrant in combination with anastrozole, a meta-analysis including FACT trial[38] and SWOG S0226 trial[39] showed that, as first-line therapy in postmenopausal women with HR-positive advanced breast cancer, the addition of fulvestrant at loading does was not efficient than anastrozole alone[40]. Another trial was conducted to explore clinical benefits among NSAIs-resistant patients, the results also showed fulvestrant plus anastrozole was not better than either fulvestrant alone or exemestane alone[41].

It has been documented that, fulvestrant could be combined with several kinds of targeted drugs, such as CDK4/6 inhibitor, PI3K inhibitor and mTOR inhibitor. Our meta-analysis indicated that, compared with fulvestrant alone, targeted therapy plus fulvestrant slightly prolonged PFS (HR = 0.77, 95%CI: 0.66–0.91) in postmenopausal patients with HR-positive advanced breast cancer. This finding is consistent with the previous published meta-analysis[42, 43]. Based on existing evidence, we could also draw forementioned conclusion in postmenopausal women with HR+/ HER- tumor (HR = 0.71, 95%CI: 0.60–0.85) or patients with ET-resistant advanced breast cancer (HR = 0.76, 95%CI: 0.63–0.92). Moreover, PIK3CA mutations represent one of the most common molecular aberrations in breast cancer[44, 45]. Several trials were failed to found a significant association between PI3K inhibitors and PIK3CA-mutant breast cancer[26, 46], which brings into question whether PIK3CA mutation are targetable in the clinic setting[47]. The present analysis showed that combination therapy would prolong PFS for postmenopausal patients with PIK3CA-mutant ctDNA (HR = 0.52, 95%CI: 0.39–0.69), whereas patients with PIK3CA mutation detected in tumor issue failed to show the significant benefit (HR = 0.70, 95%CI: 0.48–1.02). And the other endpoints concerning patients with PIK3CA–mutant cancer were too sparsely reported for a meta-analysis to be feasible.

Besides PFS, targeted drugs plus fulvestrant also slightly improved ORR for postmenopausal women with HR-positive, HER2-negative advanced breast cancer (RR = 1.78, 95%CI: 1.35–2.34). And it is also an effective choice to improve ORR in patients with measurable disease (HR = 2.35 95%CI: 1.35–4.11).

For toxicity, the currently available clinical evidence indicated that there was a weak positive correlation between the combination therapy and the incidence of adverse events (RR = 1.09, 95%CI: 1.05–1.13), and we also draw the similar conclusion for the risk of CTACAE≥3 (RR = 1.97, 95%CI: 1.49–2.60). Moreover, discontinuation due to adverse events reported in combination arms was higher than fulveatrant monotherapy (RR = 4.91, 95%CI: 3.37–7.15). Although SAEs related to drugs was significantly different between treatment groups (RR = 4.23, 95%CI: 1.62–11.03), this estimate required more robust evidence to support due to the broad range of 95%CI.

We found some limitations in this study: first, some pooled effect size, lower limit or upper limit of 95%CI were near the value 1, which might be an important factor to change significance of results in sensitivity analysis, and these relevant results in meta-analysis should be explain cautiously; second, some concerned endpoints, such as OS, adverse events related to treatment drugs were reported scarcely; third, studies of drugs targeting the same signal pathways or receptors were little, more RCTs concerning targeted therapy in combination with endocrine therapy should be conducted and published.

In conclusion, compared with fulvestrant monotherapy, targeted therapy plus fulvestrant slightly improved PFS and ORR of postmenposaul women with HR+ advanced breast cancer; besides, combination therapy also increased toxicity. To date, the majority of RCTs have not identified cancer biomarkers, which might decrease the efficacy of target drugs. Therefore, more measures should be taken to promote the progress of precision medicine for advanced breast cancer.

## Supporting information

S1 Table PRISMA 2009 Checklist. (DOC)

## References

[1] Torre LA, Bray F, Siegel RL, Ferlay J, Lortet-Tieulent J, Jemal A. Global cancer statistics, 2012. CA: a cancer journal for clinicians. 2015;65(2):87–108. 25651787 10.3322/caac.21262

[2] Harbeck N, Gnant M. Breast cancer. Lancet. 2017;389(10074):1134–1150. 10.1016/S0140-6736(16)31891-8 27865536

[3] Darby S, McGale P, Correa C, Taylor C, Arriagada R, Clarke M, et al Effect of radiotherapy after breast-conserving surgery on 10-year recurrence and 15-year breast cancer death: meta-analysis of individual patient data for 10,801 women in 17 randomised trials. Lancet. 2011;378(9804):1707–1716. 10.1016/S0140-6736(11)61629-2 22019144 PMC3254252

[4] Partridge AH, Rumble RB, Carey LA, Come SE, Davidson NE, Di Leo A, et al Chemotherapy and targeted therapy for women with human epidermal growth factor receptor 2-negative (or unknown) advanced breast cancer: American Society of Clinical Oncology Clinical Practice Guideline. Journal of clinical oncology: official journal of the American Society of Clinical Oncology. 2014; 32(29):3307–3329. 25185096 10.1200/JCO.2014.56.7479 PMC6076042

[5] Bundred N. Preclinical and clinical experience with fulvestrant (Faslodex) in postmenopausal women with hormone receptor-positive advanced breast cancer. Cancer investigation. 2005;23(2):173–181. 15813510 10.1081/cnv-50480

[6] Thill M, Liedtke C, Solomayer EF, Müller V, Janni W, Schmidt M, et al AGO Recommendations for the diagnosis and treatment of patients with advanced and metastatic breast cancer: Update 2017. Breast Care. 2017;12(3):184–191. 10.1159/000477576 28785187 PMC5527195

[7] National Comprehensive Cancer Network. NCCN Clinical Practice Guidelines in Oncology: Breast Cancer(version 4. 2017). 2018. 10.6004/jnccn.2018.0012 29523670

[8] Nathan MR, Schmid P. A Review of Fulvestrant in Breast Cancer. Oncology and therapy. 2017;5(1):17–29. 10.1007/s40487-017-0046-2 28680952 PMC5488136

[9] Wang J, Xu B, Wang W, Zhai X, Chen X. Efficacy and safety of fulvestrant in postmenopausal patients with hormone receptor-positive advanced breast cancer: a systematic literature review and meta-analysis. Breast Cancer Research and Treatment. 2018;171(3):535–544. 10.1007/s10549-018-4867-y 29974356

[10] Lee CI, Goodwin A, Wilcken N. Fulvestrant for hormone-sensitive metastatic breast cancer. Cochrane Database of Systematic Reviews. 2017;2017(1):1–59. 10.1002/14651858.CD011093.pub2 PMC6464820 28043088

[11] Osborne CK, Pippen J, Jones SE, Parker LM, Ellis M, Come S, et al Double-blind, randomized trial comparing the efficacy and tolerability of fulvestrant versus anastrozole in postmenopausal women with advanced breast cancer progressing on prior endocrine therapy: results of a North American trial. Journal of Clinical Oncology. 2002;20(16):3386–3395. 10.1200/JCO.2002.10.058 12177098

[12] Di Leo A, Jerusalem G, Petruzelka L, Torres R, Bondarenko IN, Khasanov R, et al Results of the CONFIRM phase III trial comparing fulvestrant 250 mg with fulvestrant 500 mg in postmenopausal women with estrogen receptor-positive advanced breast cancer. Journal of clinical oncology: official journal of the American Society of Clinical Oncology. 2010;28(30):4594–4600. 20855825 10.1200/JCO.2010.28.8415

[13] Polk A, Kolmos IL, Kumler I, Nielsen DL. Specific CDK4/6 inhibition in breast cancer: a systematic review of current clinical evidence. ESMO open. 2016;1(6):1–11. 10.1136/esmoopen-2016-000093 PMC5419212 28848657

[14] Fan W, Chang J, Fu P. Endocrine therapy resistance in breast cancer: Current status, possible mechanisms and overcoming strategies. Future Medicinal Chemistry. 2015;7(12):1511–1519. 10.4155/fmc.15.93 26306654 PMC5558537

[15] Murphy CG, Dickler MN. Endocrine resistance in hormone-responsive breast cancer: mechanisms and therapeutic strategies. Endocrine-Related Cancer. 2016;23(8):R337–R352. 10.1530/ERC-16-0121 27406875

[16] Baselga J, Campone M, Piccart M, Rd B, Rugo H, Sahmoud T, et al Everolimus in postmenopausal hormon-receptor-positive advanced breast cancer. New England Journal of Medicine, 2012; 366 (6):520–529. 10.1056/NEJMoa1109653 22149876 PMC5705195

[17] Turner NC, Ro J, Andre F, Loi S, Verma S, Iwata H, et al Palbociclib in Hormone-Receptor-Positive Advanced Breast Cancer. New England Journal of Medicine. 2015;373(3):209–219. 10.1056/NEJMoa1505270 26030518

[18] Tierney JF, Stewart LA, Ghersi D, Burdett S, Sydes MR. Practical methods for incorporating summary time-to-event data into meta-analysis. Trials. 2007;8. 10.1186/1745-6215-8-16 PMC1920534 17555582

[19] Hyams DM, Chan A, Oliveira C, Snyder R, Vinholes J, Audeh MW, et al Cediranib in combination with fulvestrant in hormone-sensitive metastatic breast cancer: a randomized Phase II study. Investigational new drugs. 2013; 31(5):1345–1354. 10.1007/s10637-013-9991-2 23801303

[20] Robertson JFR, Ferrero JM, Bourgeois H, Kennecke H, de Boer RH, Jacot W, et al Ganitumab with either exemestane or fulvestrant for postmenopausal women with advanced, hormone-receptor-positive breast cancer: a randomised, controlled, double-blind, phase 2 trial. Lancet Oncology. 2013;14(3):228–235. 10.1016/S1470-2045(13)70026-3 23414585

[21] Burstein HJ, Cirrincione CT, Barry WT, Chew HK, Tolaney SM, Lake DE, et al Endocrine therapy with or without inhibition of epidermal growth factor receptor and human epidermal growth factor receptor 2: a randomized, double-blind, placebo-controlled phase III trial of fulvestrant with or without lapatinib for postmenopausal women with hormone receptor-positive advanced breast cancer-CALGB 40302 (Alliance). Journal of clinical oncology: official journal of the American Society of Clinical Oncology. 2014;32(35):3959–3966. 25348000 10.1200/JCO.2014.56.7941 PMC4251959

[22] Clemons MJ, Cochrane B, Pond GR, Califaretti N, Chia SK, Dent RA, et al Randomised, phase II, placebo-controlled, trial of fulvestrant plus vandetanib in postmenopausal women with bone only or bone predominant, hormone-receptor-positive metastatic breast cancer (MBC): the OCOG ZAMBONEY study. Breast cancer research and treatment. 2014;146(1):153–162. 10.1007/s10549-014-3015-6 24924416

[23] Zaman K, Winterhalder R, Mamot C, Hasler-Strub U, Rochlitz C, Mueller A, et al Fulvestrant with or without selumetinib, a MEK 1/2 inhibitor, in breast cancer progressing after aromatase inhibitor therapy: a multicentre randomised placebo-controlled double-blind phase II trial, SAKK 21/08. European journal of cancer. 2015; 51(10):1212–1220. 10.1016/j.ejca.2015.03.016 25892646

[24] Adelson K, Ramaswamy B, Sparano JA, Christos PJ, Wright JJ, Raptis G, et al Randomized phase II trial of fulvestrant alone or in combination with bortezomib in hormone receptor-positive metastatic breast cancer resistant to aromatase inhibitors: a New York Cancer Consortium trial. NPJ breast cancer. 2016;2:16037 10.1038/npjbcancer.2016.37 28721390 PMC5515340

[25] Cristofanilli M, Turner NC, Bondarenko I, Ro J, Im SA, Masuda N, et al Fulvestrant plus palbociclib versus fulvestrant plus placebo for treatment of hormone-receptor-positive, HER2-negative metastatic breast cancer that progressed on previous endocrine therapy (PALOMA-3): final analysis of the multicentre, double-blind, phase 3 randomised controlled trial. The Lancet Oncology. 2016;17(4):425–439. 10.1016/S1470-2045(15)00613-0 26947331

[26] Krop IE, Mayer IA, Ganju V, Dickler M, Johnston S, Morales SN, et al Pictilisib for oestrogen receptor-positive, aromatase inhibitor-resistant, advanced or metastatic breast cancer (FERGI): a randomised, double-blind, placebo-controlled, phase 2 trial. Lancet Oncology. 2016;17(6):811–821. 10.1016/S1470-2045(16)00106-6 27155741 PMC5524539

[27] Baselga J, Im SA, Iwata H, Cortes J, De Laurentiis M, Jiang Z, et al Buparlisib plus fulvestrant versus placebo plus fulvestrant in postmenopausal, hormone receptor-positive, HER2-negative, advanced breast cancer (BELLE-2): a randomised, double-blind, placebo-controlled, phase 3 trial. The Lancet Oncology. 2017;18(7):904–916. 10.1016/S1470-2045(17)30376-5 28576675 PMC5549667

[28] Loibl S, Turner NC, Ro J, Cristofanilli M, Iwata H, Im SA, et al Palbociclib Combined with Fulvestrant in Premenopausal Women with Advanced Breast Cancer and Prior Progression on Endocrine Therapy: PALOMA-3 Results. Oncologist. 2017;22(9):1028–1038. 10.1634/theoncologist.2017-0072 28652278 PMC5599195

[29] Musolino A, Campone M, Neven P, Denduluri N, Barrios CH, Cortes J, et al Phase II, randomized, placebo-controlled study of dovitinib in combination with fulvestrant in postmenopausal patients with HR(+), HER2(-) breast cancer that had progressed during or after prior endocrine therapy. Breast cancer research. 2017;19(1):18 10.1186/s13058-017-0807-8 28183331 PMC5301372

[30] Sledge GW Jr., Toi M, Neven P, Sohn J, Inoue K, Pivot X, et al MONARCH 2: Abemaciclib in Combination With Fulvestrant in Women With HR+/HER2- Advanced Breast Cancer Who Had Progressed While Receiving Endocrine Therapy. Journal of clinical oncology: official journal of the American Society of Clinical Oncology. 2017;35(25):2875–2884. 28580882 10.1200/JCO.2017.73.7585

[31] Di Leo A, Johnston S, Lee KS, Ciruelos E, Lonning PE, Janni W, et al Buparlisib plus fulvestrant in postmenopausal women with hormone-receptor-positive, HER2-negative, advanced breast cancer progressing on or after mTOR inhibition (BELLE-3): a randomised, double-blind, placebo-controlled, phase 3 trial. The Lancet Oncology. 2018;19(1):87–100. 10.1016/S1470-2045(17)30688-5 29223745

[32] Cardoso F, Costa A, Senkus E, Aapro M, André F, Barrios CH, et al 3rd ESO-ESMO International Consensus Guidelines for Advanced Breast Cancer (ABC 3). Annals of Oncology. 2017;28(1):16–33. 10.1093/annonc/mdw544 28177437 PMC5378224

[33] Davies C, Godwin J, Gray JR, Clarke M, Darby S, McGale P, Wang Y. C., Peto R, et al Relevance of breast cancer hormone receptors and other factors to the efficacy of adjuvant tamoxifen: patient-level meta-analysis of randomised trials. Lancet. 2011;378(9793):771–784. 10.1016/S0140-6736(11)60993-8 21802721 PMC3163848

[34] Cepa M, Vaz C. Management of bone loss in postmenopausal breast cancer patients treated with aromatase inhibitors. Acta Reumatol Port. 2015;40(4):323–330. 26922195

[35] De Marchi T, Foekens JA, Umar A, Martens JWM. Endocrine therapy resistance in estrogen receptor (ER)-positive breast cancer. Drug Discovery Today. 2016;21 (7):1181–1188. 10.1016/j.drudis.2016.05.012 27233379

[36] Fan WM, Chang JJ, Fu PF. Endocrine therapy resistance in breast cancer: current status, possible mechanisms and overcoming strategies. Future Medicinal Chemistry. 2015;7(12):1511–1519. 10.4155/fmc.15.93 26306654 PMC5558537

[37] Di Leo A, Jerusalem G, Petruzelka L, Torres R, Bondarenko IN, Khasanov R, et al Final overall survival: fulvestrant 500 mg vs 250 mg in the randomized CONFIRM trial. Journal of the National Cancer Institute. 2014;106(1):djt337 10.1093/jnci/djt337 24317176 PMC3906991

[38] Bergh J, Jonsson P-E, Lidbrink EK, Trudeau M, Eiermann W, Brattstrom D, et al FACT: An Open-Label Randomized Phase III Study of Fulvestrant and Anastrozole in Combination Compared With Anastrozole Alone As First-Line Therapy for Patients With Receptor-Positive Postmenopausal Breast Cancer. Journal of Clinical Oncology. 2012;30(16):1919–1925. 10.1200/JCO.2011.38.1095 22370325

[39] Mehta RS, Barlow WE, Albain KS, Vandenberg TA, Dakhil SR, Tirumali NR, et al Combination anastrozole and fulvestrant in metastatic breast cancer. The New England journal of medicine. 2012;367(5):435–444. 10.1056/NEJMoa1201622 22853014 PMC3951300

[40] Tan PS, Haaland B, Montero AJ, Lopes G. A meta-analysis of anastrozole in combination with fulvestrant in the first line treatment of hormone receptor positive advanced breast cancer. Breast cancer research and treatment. 2013;138(3):961–965. 10.1007/s10549-013-2495-0 23542955

[41] Johnston SR, Kilburn LS, Ellis P, Dodwell D, Cameron D, Hayward L, et al Fulvestrant plus anastrozole or placebo versus exemestane alone after progression on non-steroidal aromatase inhibitors in postmenopausal patients with hormone-receptor-positive locally advanced or metastatic breast cancer (SoFEA): a composite, multicentre, phase 3 randomised trial. The Lancet Oncology. 2013;14(10):989–998. 10.1016/S1470-2045(13)70322-X 23902874

[42] Mayer IA, O’Regan R, Kornblum NS, Blackwell KL. Targeted combination therapy with fulvestrant (FUL) for second-line (2L) treatment of hormone receptor-positive (HR+) advanced breast cancer (ABC). Journal of Clinical Oncology. 2017;35(15).

[43] Lin WZ, Xu QN, Wang HB, Li XY. Fulvestrant plus targeted agents versus fulvestrant alone for treatment of hormone-receptor positive advanced breast cancer progressed on previous endocrine therapy: a meta-analysis of randomized controlled trials. Breast Cancer. 2017;24(3):345–352. 10.1007/s12282-017-0770-3 28324247

[44] Azizi Tabesh G, Izadi P, Fereidooni F, Emami Razavi AN, Tavakkoly Bazzaz J. The High Frequency of PIK3CA Mutations in Iranian Breast Cancer Patients. Cancer investigation. 2017;35(1):36–42. 10.1080/07357907.2016.1247455 27901576

[45] Elwy F, Helwa R, El Leithy AA, Shehab El din Z, Assem MM, Hassan NH. PIK3CA mutations in HER2-positive Breast Cancer Patients; Frequency and Clinicopathological Perspective in Egyptian Patients. Asian Pacific journal of cancer prevention. 2017;18(1):57–64. doi: 10.22034/APJCP.2017.18.1.57 28240010 10.22034/APJCP.2017.18.1.57 PMC5563120

[46] Moynahan ME, Chen D, He W, Sung P, Samoila A, You D, et al Correlation between PIK3CA mutations in cell-free DNA and everolimus efficacy in HR+, HER2(-) advanced breast cancer: results from BOLERO-2. British Journal Of Cancer. 2017;116(6):726–730. 10.1038/bjc.2017.25 28183140 PMC5355930

[47] Chopra N, Turner NC. Targeting PIK3CA-mutant advanced breast cancer in the clinical setting. The Lancet Oncology. 2017;18(7):842–843. 10.1016/S1470-2045(17)30430-8 28576676

